# Geographical patterns and determinants in plant reproductive phenology duration

**DOI:** 10.3389/fpls.2023.1199316

**Published:** 2023-06-15

**Authors:** Xinyang Wang, Xavier Morin, Jian Zhang, Guoke Chen, Lingfeng Mao, Yuheng Chen, Zhuqiu Song, Yanjun Du, Keping Ma

**Affiliations:** ^1^ Key Laboratory of Genetics and Germplasm Innovation of Tropical Special Forest Trees and Ornamental Plants (Ministry of Education), College of Forestry, Hainan University, Haikou, China; ^2^ Centre d'Écologie Fonctionnelle et Évolutive (CEFE), Centre National de la Recherche Scientifique (CNRS), University of Montpellier, Ecole Pratique des Hautes Etudes (EPHE), Institut de recherche pour le Développement (IRD), Univ. Paul Valéry Montpellier 3, Montpellier, France; ^3^ School of Ecological and Environmental Sciences, East China Normal University, Shanghai, China; ^4^ State Key Laboratory of Vegetation and Environmental Change, Institute of Botany, Chinese Academy of Sciences, Beijing, China; ^5^ Co-Innovation Center for Sustainable Forestry in Southern China, College of Biology and the Environment, Nanjing Forestry University, Nanjing, China; ^6^ Key Laboratory of Plant Resources Conservation and Sustainable Utilization, South China Botanical Garden, Chinese Academy of Sciences, Guangzhou, China

**Keywords:** flowering phenology duration, fruiting phenology duration, latitudinal gradient, longitude pattern, life forms

## Abstract

Biodiversity is and always has been an important issue in ecological research. Biodiversity can reflect niche partitioning among species at several spatial and temporal scales and is generally highest in the tropics. One theory to explain it is that low-latitude tropical ecosystems are dominated by species that are generally only distributed over a narrow area. This principle is known as Rapoport’s rule. One previously unconsidered extension of Rapoport’s rule may be reproductive phenology, where variation in flowering and fruiting length may reflect a temporal range. Herein, we collected reproductive phenology data for more than 20,000 species covering almost all angiosperm species in China. We used a random forest model to quantify the relative role of seven environmental factors on the duration of reproductive phenology. Our results showed that the duration of reproductive phenology decreased with latitude, although there was no obvious change across longitudes. Latitude explained more of the variation in the duration of flowering and fruiting phases in woody plants than in herbaceous plants. Mean annual temperature and the length of the growing season strongly influenced the phenology of herbaceous plants, and average winter temperature and temperature seasonality were important drivers of woody plant phenology. Our result suggests the flowering period of woody plants is sensitive to temperature seasonality, while it does not influence herbaceous plants. By extending Rapoport’s rule to consider the distribution of species in time as well as space, we have provided a novel insight into the mechanisms of maintaining high levels of diversity in low-latitude forests.

## 1 Introduction

Phenology generally refers to the timing of flowering and fruiting in plants, important drivers of the distribution of species (Chuine, 2010). For example, the distribution range of species is often determined by the limitation of flowering phenology. For example, an environment may be too warm for a species, wherein a lack of low temperature prevents the break of seed dormancy. Conversely, early or premature flowering species may suffer frost damage (Morin et al., 2007). A large number of studies have shown that the species ranges of vascular plant species are significantly changing because of global warming (Kiritani, 2006; Subedi et al., 2015). The degree to which species range shifts will depend on the degree to which population growth rates are sensitive to changes in reproduction and the degree to which duration affects reproductive fitness (Iler et al., 2019). Consequently, it is necessary to understand how plants may respond to climate change. In particular, this research may be necessary for the formulation of endangered species reduced to smaller distribution ranges by the changes in climate (Bykova et al., 2012).

George C. Stevens christened the decline in the geographic range of species from high to low latitudes as Rapoport’s rule (Stevens, 1989). Colwell et al. further considered the relationship between species richness and range size and believed that Rapoport’s rule was only valid when the range size was larger and the overlap was small (Colwell & Hurtt, 1994). For example, several tropical species have wide spatial ranges—but this “inverse Rapoport’s rule” is only possible because these species broadly overlap in their tolerance for the environment (a homogenously hot and humid jungle) (Tomašových et al., 2015). Despite these exceptions, the rule has been shown to effectively predict species distributions in a number of systems. For example, the rule is considered valid in the high latitudes of the Northern Hemisphere. Possible explanations for Rapoport’s rule include climate variability (Dobzhansky, 1950), extreme climates (Pither, 2003), and varying degrees of extinction or glacier history (Jansson, 2003). However, previous studies on Rapoport’s rule of plant species have not considered an important dimension of species distribution—the timing of reproduction (Zhou et al., 2019). It has yet to be established if Rapoport’s rule could be applied to temporal ranges, such as the duration of reproductive phenology (i.e., the timing of flowering and fruiting).

Climate drives plant reproductive phenology, and temperature and precipitation trigger the growth and development of many reproductive structures (Rathcke & Lacey, 1985; Høye et al., 2007). Considering that the climate environment changes with the geographical gradient, Ting et al. (2008) concluded that the duration of the fruiting period gradually shortened with increasing latitude (Ting et al., 2008). However, ignoring longitudinal changes in climate and geography that influence the duration of reproductive phenology may in turn create large uncertainties in our study of the influence of latitude. This premise, that longitudinal climate variation influences phenology, is consistent with recent work on the phenology of common alder (Ziello et al., 2009). This makes intuitive sense. For example, the average annual rainfall in China gradually decreases with decreasing east longitude and increasing northern latitude (Xu & Zhang, 2017), which limits the duration of photosynthesis (Huxman et al., 2004). This in turn may shorten the duration of reproductive phenology. Therefore, a comprehensive description of the spatial patterns of the duration of reproductive phenology with latitude and longitude is required.

The effect of climate on plant reproductive phenology can vary across life forms and may vary predictably across functional types (Du et al., 2020). Woody plants usually have larger seeds than their herbaceous counterparts (Leishman et al., 2000; Moles & Westoby, 2006), and the longer development time of large seeds directly limits the fruit-setting time, which may explain the earlier flowering period of woody plants (Chuine, 2010). Woody and herbaceous species also have different strategies to ensure access to light and response to seasonal environments (Westoby et al., 2002). This may in turn influence reproduction, and the flowering and fruiting of herbaceous plants and woody plants both show a strong seasonal pattern, though they are distinctly different (Batalha & Martins, 2004). Most herbaceous perennials exhibit within-individual flowering asynchrony, requiring minimal resource consumption, while most woody plants exhibit within-individual flowering synchrony, requiring considerable resource consumption (Crimmins et al., 2013). Therefore, the key environmental factors influencing phenology in different life forms may also be different.

Relying on phenological data collected from >20,000 species of angiosperm flora in China, we analyzed the large-scale geographic pattern of phenological duration in flowering and fruiting phenology, as well as the climatic factors influencing these patterns. Mainland China has diverse climatic regions, including tropical, subtropical, and temperate climatic regions from south to north. It thus provides a large-scale natural experiment to study the phenological patterns of plants. Specifically, we aimed at addressing the following questions:

1. How does the duration of reproductive phenology change with latitude and longitude? We predicted that as the latitude increases, the duration of the reproductive phenology of species will gradually decrease. We also predict a gradual decrease in reproductive “range” as we move from east to west into increasingly wetter forests.
2. Will any observed patterns in variation in reproductive phenology duration be different in woody and herbaceous plants? We predict that both woody and herbaceous plants will show a decrease in the flowering period, but we anticipate a difference in the slope and correlation of that relationship between woody and herbaceous plants.
3. Finally, does climate influence the flowering period of woody and herbaceous plants in a similar way, or are they influenced by distinctly different factors in the local climate? We hypothesize there are large differences between herbaceous and woody plants.

## 2 Materials and methods

### 2.1 Species distribution and phenology data

The species distribution data come from the database of the distribution of Chinese vascular plants, which describes more than 6 million specimens (Xu et al., 2018). All records in this database include geographic information describing the local province. In this way, the database contains the information necessary to calculate the length of the flowering period for all angiosperm species recorded in each province. For each province, if a species is found in that province, we scored it as an existing species. For populations of the same species in different provinces, their phenological record data are often different. We recorded the start and end times of the reproductive phenology period for all the angiosperms in each province and obtained flowering and fruiting phenology duration data from the local flora of 28 provinces. We calculated the average reproductive phenology duration at the provincial level because we can only obtain provincial phenological data, as county-level phenological data currently do not exist. Moreover, we stress that because of the inconsistency of recording years, the average of all species’ phenological duration in each province’s data can only represent the average annual time phenology data.

For each species in a province, we collected five pieces of information: the earliest and latest dates of flowering, the earliest and latest dates of fruiting, and life form (i.e., woody or herbaceous). For example, we recorded that *Lithocarpus henryi* blooms from August (the first day is day 213) to October (the last day is day 304), so the flowering duration in days is 304 − 213 = 91 days. For species whose phenological periods extend across years, we used the sum of their respective durations over 2 years as their actual durations.

In total, we recorded the flowering dates of 24,304 species of plants belonging to 2,633 genera and 228 families. Of these species, 61% (15,043) are herbaceous plants and 39% (9,261) are woody plants. We recorded the fruiting dates of 18,792 species belonging to 222 families and 2,370 genera (many species such as orchids and bamboo only have flowering phenology records but no fruiting phenology records). Of these species, 56% (10,580) are herbaceous plants and 44% (8,212) are woody plants.

### 2.2 Climatic and geographic data

We selected six potential environmental factors from the WorldClim database (Hijmans et al., 2005) that are generally considered to have an impact on plant phenology as non-biological factors for statistical analysis. To ensure a balance of environmental factors, we have selected three temperature-related environmental factors and three precipitation-related environmental factors that are related to the reproductive phenology period. The full name and abbreviation of the environmental variable used are as follows: mean annual temperature (MAT), temperature seasonality (TS; standard deviation within monthly values), mean temperature of coldest quarter (MTC), mean annual precipitation (MAP), precipitation seasonality (PS; standard deviation within monthly values), and precipitation of driest quarter (MPD). Environmental data extracted from WorldClim are considered to represent ~1 km^2^. This is typical of that of the climate models used in the studies (Challinor et al., 2015).

Accumulated temperature is one way to measure how heat affects plant growth and distribution (Xu et al., 2021). We generated accumulated temperature ≥0°C (Clark et al., 2014) using data from China’s Meteorological background dataset (Xu & Zhang, 2017). Accumulated temperature (sum of daily mean temperatures ≥ 0°C) has a spatial resolution of 500 m and can be obtained from the Resource and Environment Science and Data Center (RESDC; https://www.resdc.cn/).

The mean value of all environmental factors for each province was calculated using ArcGIS (Version 10.2, ESRI, 2016). Previous studies underscore the tight connection between phenology and local environmental conditions changes within large-scale provinces in Xinjiang, Tibet, and Qinghai (Li C. et al., 2021; Shen et al., 2022).

### 2.3 Data analyses

We conducted statistical analyses on each of three plant groups: 1) total (all species), 2) woody species, and 3) herbaceous species. To evaluate the relative effects of latitude and longitude on the average flowering and fruiting phenology durations of all species in each region, we used a linear model (LM). We used ArcGIS software (ArcGIS 10.3) to label the provinces of China with colors based on the average duration of the phenological period in each province. We regressed flowering duration data and fruiting duration data against the seven environmental factors using random forest (RF) regression to detect which variable can explain the most variance in flowering and fruiting durations. We chose this method because it does not require strict data assumptions and can better handle multicollinearity and non-linear relationships, which troubles most traditional methods like generalized linear models (GLMs) (Breiman, 2001; Feng et al., 2016). The random forest model was built using the randomForest package in R (R Core Team, 2020). We generated variable indexes and partial dependence graphs to demonstrate the importance of the explanatory variables on the response variables (Auret and Aldrich, 2012). We selected the most critical factor based on the value of the MSE (mean square error) index.

Random forests (Skidmore et al., 1996) use model averaging to generate predictions. The fit of each decision tree is evaluated using randomly selected cases (one-third of the data) that are retained during the construction of the forest. When the data for the predictor are randomly arranged, the importance of each predictor will be determined by evaluating the decrease in prediction accuracy (Delgado‐Baquerizo et al., 2016). This allows the user to avoid problems like autocorrelation and variable collinearity. Its reliability has been confirmed in a number of previous studies (Bradter et al., 2013; Fox et al., 2017).

## 3 Results

### 3.1 Geographical patterns of flowering and fruiting phenology durations

**Figure 1:**
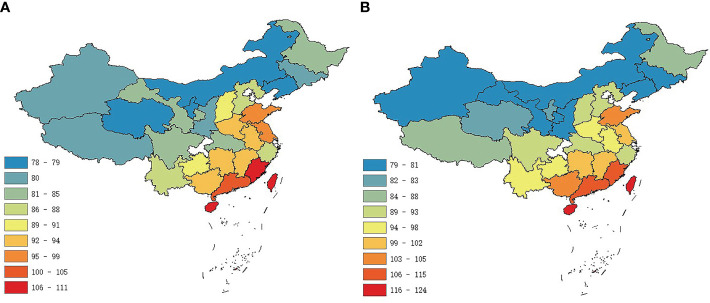
Geographical variation in the durations of reproductive phenology of angiosperms in China estimated for provincial level. Colors represent the mean phenology duration (in days) for **(A)** flowering and **(B)** fruiting.

There were obvious latitudinal patterns in the case of flowering duration (**Figure 1A**) and fruiting duration (**Figure 1B**). The durations of the reproductive phenology of plants, for both flowering phenology and fruiting phenology, were highly correlated with latitude (flowering duration for total: R^2^ = 0.51, C = −0.72; fruiting duration for total: R^2^ = 0.67, C = −0.82) (**Table 1**). We found no relationship with longitude (R^2^ = 0.11 and 0.06, *p* = 0.09 and 0.21, respectively) (**Table 1**).

**Table 1:** Regression results between flowering and fruiting durations for total, herbaceous, and woody plants in each province in China with latitude and longitude.

		Flowering period			Fruiting period		
		Total	Herb	Woody	Total	Herb	Woody
Latitude	R^2^	0.51	0.63	0.81	0.67	0.56	0.83
	*p*	<0.001	<0.001	<0.001	<0.001	<0.001	<0.001
	Slope	−0.61	−0.84	−1.17	−0.72	−1.09	−1.03
Longitude	R^2^	0.11	0.10	0.01	0.06	0.11	0
	*p*	0.09	0.10	0.72	0.21	0.08	0.80
	Slope	0.31	0.50	−0.07	0.31	0.44	0.07

### 3.2 Flowering duration and life form

**Figure 2:**
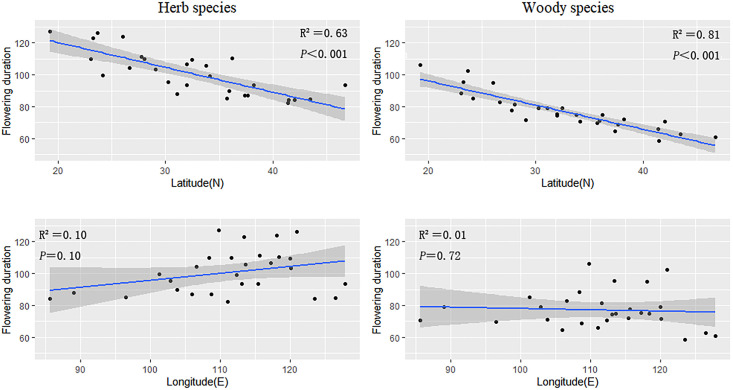
The regression results of the average flowering duration of herbaceous and woody plant groups in every province against latitude and longitude, respectively.

Geographical patterns in the flowering duration were relatively similar for herbaceous and woody plants. The flowering duration of herbaceous and woody plants decreased as latitude increased (slope = −0.84 and −1.17, respectively) (**Figure 2**; **Table 1**). The flowering durations of herb and woody plants were highly correlated with latitude (herb species, R^2^ = 0.63; woody species, R^2^ = 0.81) (**Figure 2**; **Table 1**), but not with longitude.

### 3.3 Fruiting duration and life form

**Figure 3:**
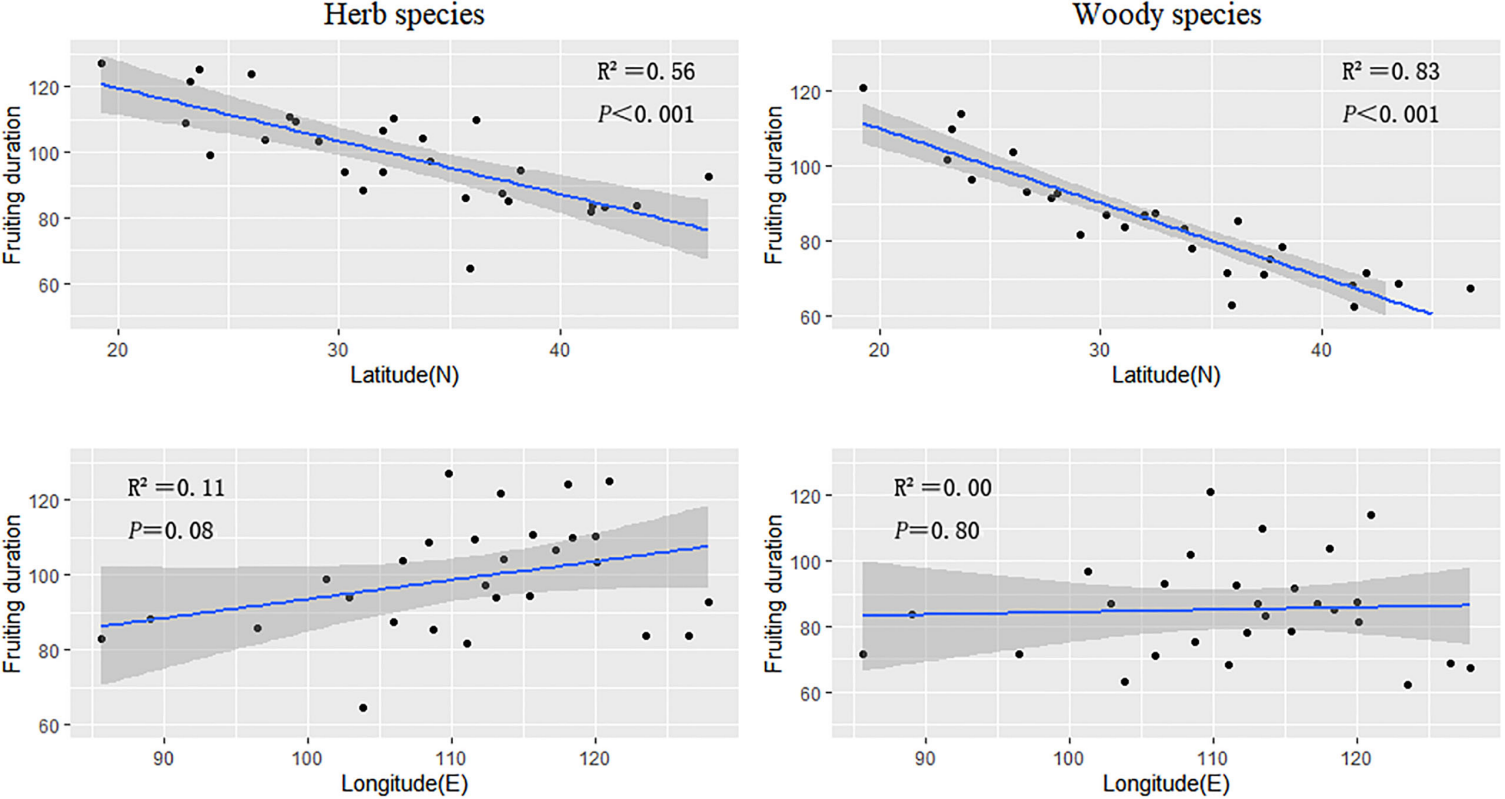
The regression results of the average fruiting duration of herbaceous and woody plant groups in every province against latitude and longitude.

The fruiting durations of herbaceous and woody plants also decreased as latitude increased (slope = −1.09 and −1.03, respectively) (**Figure 3**; **Table 1**). The fruiting period was highly correlated with latitude (herb species, R^2^ = 0.56; woody species, R^2^ = 0.83).

### 3.4 Climatic correlates of flowering and fruiting durations

**Figure 4:**
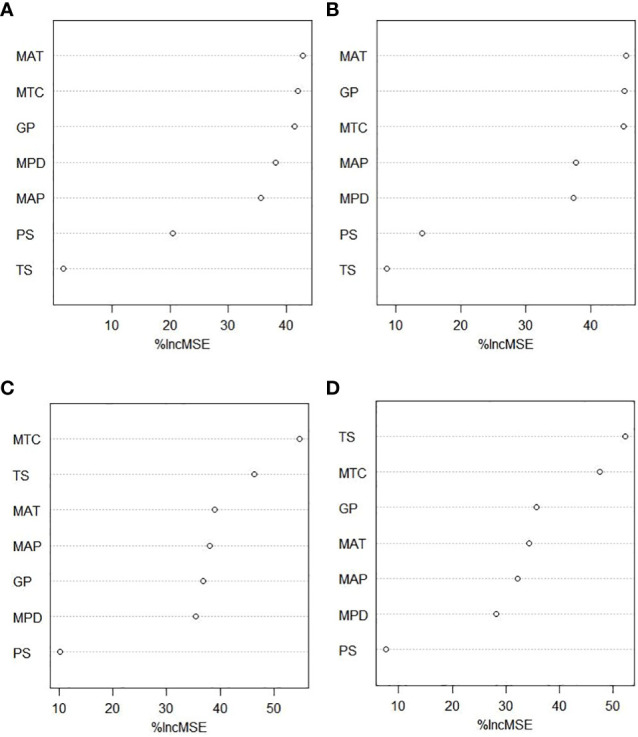
The importance of environmental factors. **(A)** Herbaceous fruiting duration. **(B)** Herbaceous flowering duration. **(C)** Woody fruiting duration. **(D)** Woody flowering duration. Mean annual temperature (MAT), temperature seasonality (TS), mean temperature of coldest quarter (MTC), mean annual precipitation (MAP), precipitation seasonality (PS), precipitation of driest quarter (MPD), and the length of growing season (GP) to reproductive phenological duration. The plot shows the variable importance measured as the increased mean square error (%IncMSE), which represents the deterioration of the predictive ability of the model when each predictor is replaced in turn by random noise. Higher %IncMSE indicates greater variable importance.

As shown in **Figure 4**, mean annual temperature (MSE = 16.72) and the length of the growing season (MSE = 16.20) were the most important determinants of the reproductive phenology duration for herbaceous plants (**Figures 4A, B**). However, for woody plants, the length of the growing season was not so important, and instead, the mean temperature of the coldest quarter (MSE = 16.72) was the most important factor, followed by the temperature seasonality (MSE = 15.06) (**Figures 4C, D**). It should be emphasized that the flowering and fruiting durations of herbaceous plants and woody plants were both consistently correlated with environmental factors.

## 4 Discussion

### 4.1 Spatial patterns of reproductive phenology duration

Rapoport’s rule would suggest that the duration of reproductive phenology would be positively correlated with latitude. However, we observed plant species flowered longer in warmer regions, a result supported by several other studies (Ting et al., 2008; Li D. et al., 2021). Reproductive phenology can be affected by a host of environmental factors, such as photoperiod, temperature, and rainfall, all of which are closely related to geographic location (Gerst et al., 2017). The stronger seasonal restrictions that climate places on plant growth and the availability of pollinators (for insect-pollinated species) may concentrate the reproductive phenology period of species in high latitudes into a relatively short seasonal period (Rathcke & Lacey, 1985; Diaz et al., 1994). Plant communities at lower latitudes are generally considered to have higher primary productivity and annual temperature (Gillman et al., 2015). Increasing annual temperatures drive the earlier onset of flowering and fruiting but only drive earlier offset time in minority species located in low latitudes (Li D. et al., 2021).

Rapoport’s rule would also suggest that living in low latitudes requires having a smaller distribution range, that is, a narrower space niche width. It is possible environmental pressure drives the upper latitude limit, while biological interactions are more important at lower latitudes, although there is still little evidence to support this scenario (Sax, 2001). Tropical regions typically house large assemblages of fruit-eating animals, many of which are non-migratory. Extended fruiting seasons in the tropics might therefore facilitate seed dispersal by sedentary frugivores. Conversely, many temperate regions receive massive annual influxes of migratory fruit-eating birds (Herrera, 1998). Shorter fruiting seasons, which coincide with autumn migrations of avian seed dispersers that winter in the tropics, may facilitate the spread of seeds over larger distances (Thompson & Willson, 1979). One of the main constraints on the duration of the flowering period is the appropriate climate, and the other is mainly from the competition for pollinators. The higher species abundance in low-latitude areas may lead to more competitors of similar pollination during the flowering period, and the probability of successful pollination is lower. Therefore, only plants with a longer flowering duration can survive in the tropics. Klaus Rohde’s research on marine invertebrates also shows that the diffusion ability of species in low latitudes may be greater than in high latitudes, lending further support to this premise (Stauffer & Rohde, 2006).

No obvious longitudinal pattern was observed across the woody, herb, and total plant taxa that we surveyed, potentially suggesting that the presumed longitudinal precipitation gradient did not influence reproductive phenology within the study area (Xu & Zhang, 2017).

### 4.2 Spatial patterns of herbaceous and woody plant phenology

In the present study, the reproductive phenology durations of woody and herb plants all showed a significant latitudinal pattern. However, latitude explained more of the numerical variation in the durations of flowering and fruiting phases in woody plants than in herbaceous plants. There are several reasons this may be the case. For example, the flower buds of trees and shrubs are directly exposed to ambient air, so they can better track temperature changes, both year-to-year and long-term warming trends (Heberling et al., 2019). This may be because, over time, trees may shade wildflowers and reduce their full sun hours in early spring. Second, once the ambient temperature approaches its optimal physiological range, these species can rapidly activate reproductive tissue without first producing maintenance photosynthetic tissue present in many herbal species (Du et al., 2020). Low-growing herbaceous plants are also climate-adaptive, but due to their smaller size, their response to warming trends is buffered by near-earth climate conditions (Hidalgo-Galvez et al., 2022) and limited light quality.

### 4.3 Which factors play a key role in the spatial patterning of reproductive phenology?

Consistently with our hypothesis, we found that the degree of correlation between reproductive phenology and climatic factors depends on the plant functional type and that there is a significant difference between herbaceous and woody species. Within each functional type, however, the correlation between the durations of the flowering/fruiting period and environmental factors was always highly consistent. Almost all seven climatic factors have some impact on the duration of reproductive phenology, albeit in different ways. The average annual temperature, the coldest season temperature, and the length of the growing season are the three most important climatic factors for herbaceous plants, whereas the temperature seasonality and the coldest season temperature are the two most important climatic factors for woody plants.

We emphasize that our results suggest herbaceous plants are not sensitive to seasonal precipitation and temperature seasonality and that woody plants are insensitive to temperature seasonality (Du et al., 2020). This may be because of broad ecological differences across these functional types. For example, frost damage greatly limits the start time of the reproductive phenology of woody plants. Larger shrubs and trees may intercept more light than understory plants or buffer the effects of climate change, preventing understory plants (mostly herbaceous species) from being affected by frost (re: temperature seasonality) to the same extent.

Another significant difference is that the duration of the growth period is one of the most important factors influencing the duration of the phenological period for herbaceous plants, but not for woody plants. In most species, the start of the growth phase limits the onset of flowering, while its end sets a hard boundary for fruiting (indirectly, flowering) since seed production must be completed before plant growth stops (Morin & Chuine, 2006). Larger seeds and longer fruit development times in woody plants may account for this difference. This may have also been observed because most woody plants are perennials. Before the start of their growing season, they may rely on reserves previously stored. They also would have a well-developed root system, allowing them to absorb water and other required substances.

To our knowledge, this is the first study to investigate the dynamics of the durations of flowering and fruiting phenology along latitude and longitude at the same time. Our results suggest that plants in higher latitudes tend to have shorter reproductive phenology durations, meaning they have a shorter window of time to produce seeds and disperse pollen (Segrestin et al., 2018). This has implications for the success of plant populations in these areas (Kameyama & Kudo, 2009). For example, if warmer temperatures cause an earlier onset of spring, plants may begin their reproductive stages earlier than usual, but if this occurs before pollinators are active, there may be a mismatch between the timing of flowering and the availability of pollinators, which can lead to reduced reproductive success (Kudo & Ida, 2013). This could ultimately result in range shifts (Chuine & Beaubien, 2001). Moreover, changes in the timing and duration of flowering and fruiting may disrupt interactions between plants and their pollinators, herbivores, and pathogens, reducing fitness at multiple trophic levels (Kudo & Cooper, 2019).

## Data availability statement

The original contributions presented in the study are included in the article/supplementary material. Further inquiries can be directed to the corresponding author.

## Author contributions

XW and YD conceived the ideas. All authors contributed to generating the data. XW analyzed the data. XW, XM, JZ, YD, and KM led the writing of the manuscript. All authors commented on earlier versions of the manuscript. All authors contributed to the article and approved the submitted version.
